# Inclusion Complexation of Remdesivir with Cyclodextrins: A Comprehensive Review on Combating Coronavirus Resistance—Current State and Future Perspectives

**DOI:** 10.3390/molecules29194782

**Published:** 2024-10-09

**Authors:** Arumugam Anitha, Rajaram Rajamohan, Moorthiraman Murugan, Jeong Hyun Seo

**Affiliations:** 1 PG and Research Department of Chemistry, Government Arts College, Chidambaram 608 102, Tamil Nadu, India; anithamuruganchem@gmail.com; 2 School of Chemical Engineering, Yeungnam University, Gyeongsan 38541, Republic of Korea; 3 Department of Chemistry, IFET College of Engineering, Villupuram 605 108, Tamil Nadu, India; m_murugan79@rediffmail.com

**Keywords:** remdesivir, cyclodextrins, inclusion complexes, COVID-19

## Abstract

Cyclodextrin (CD) derivatives have gained significant attention in biomedical applications due to their remarkable biocompatibility, unique inclusion capabilities, and potential for functionalization. This review focuses on recent advancements in CD-based assemblies, specifically their role in improving drug delivery, emphasizing remdesivir (RMD). The review introduces CD materials and their versatile applications in self-assembly and supramolecular assembly. CD materials offer immense potential for designing drug delivery systems with enhanced activity. Their inherent inclusion capabilities enable the encapsulation of diverse therapeutic agents, including RMD, resulting in improved solubility, stability, and bioavailability. The recent advances in CD-based assemblies, focusing on their integration with RMD have been concentrated here. Various strategies for constructing these assemblies are discussed, including physical encapsulation, covalent conjugation, and surface functionalization techniques. Furthermore, exploring future directions in these fields has also been provided. Ongoing research efforts are directed toward developing novel CD derivatives with enhanced properties, such as increased encapsulation efficiency and improved release kinetics. Moreover, the integration of CD-based assemblies with advanced technologies such as nanomedicine and gene therapy holds tremendous promise for personalized medicine and precision therapeutics

## 1. Introduction 

As many as 20% to 40% of critically ill patients experience Acute Kidney Disease (AKI) due to severe COVID-19 infection, and the End Stage Kidney Disease (ESKD) population is at higher risk for exposure and severe infection. However, many patients will not be treated with a potentially beneficial agent like remdesivir (RMD). The purpose of this article is to review what is already known about RMD and its potential risks in patients with impaired kidney function. New antiviral drugs are needed every year to treat viral infections, which kill millions of people. Such drugs should be non-toxic and irreversibly inhibit virus replication. We are not aware of any virucidal molecules that are not cytotoxic. Viruses that infect food crops and livestock, such as Human immunodeficiency virus (HIV), Ebola, and Zika virus (ZIKV), can adversely affect society on several levels. It is imperative to administer drugs that prevent viral replication and aid the immune system in fighting an infection if prevention is not possible. Current intracellular antivirals are very few in number, have permeability and toxicity problems, and are virus-specific [1], as well as having a reversible effect called virustatic [2,3,4].

### 1.1. Treatment Strategies

In recent years, several antiviral medications previously used to treat viruses like Ebola and HIV have been repurposed to treat COVID-19. However, there is no licensed treatment available for the viruses. Symptoms of the disease are relieved with medication. To preserve lives, innovative medicines need to be created. A global team of scientists, physicians, and governments is working hard to develop and implement life-saving treatments for COVID-19. The COVID-19 pandemic has necessitated developing and implementing various treatment strategies to combat the virus and alleviate the severity of symptoms. While specific treatments may vary based on individual patient factors and evolving scientific understanding, the following strategies have been widely used: Supportive Care, Antiviral Therapies, Monoclonal Antibodies, Corticosteroids, Immunomodulatory Drugs, Anticoagulation Therapy, and Vaccination.

### 1.2. Antiviral Drugs with Current Issues

There are promising outcomes from repurposing antiviral combinations to treat COVID-19. Antiviral medication development may, however, be hampered by formulation issues, particularly the active compound’s poor aqueous solubility [5]. Antiviral drugs used to treat coronavirus, specifically SARS-CoV-2 (the virus responsible for COVID-19), have been critical in managing severe cases and reducing mortality rates. The drugs primarily focus on inhibiting viral replication, disrupting the virus’s life cycle, or modulating host responses to limit disease progression (Table 1).

Unlike some of the specified above antivirals, RMD has a low potential for drug–drug interactions. In the pivotal ACTT-1 trial in hospitalized patients with COVID-19, daily intravenous infusions of RMD significantly reduced the time to recovery relative to placebo [14,15]. With this in our mind, we made this review and completely focused on the RMD and its recent advancements in COVID-19 in the form of cyclodextrin-based complexes.

### 1.3. Introduction to RMD

In several steps, ribose derivatives can be used to produce RMD. In a study from Gilead Sciences, Chun and coauthors developed a way of synthesizing RMD from ribose derivatives [16,17]. 2-Ethylbutyl((*S*)-(((2*R*,3*S*,4*R*,5*R*)-5-(4-aminopyrrolo[2,1-f][1,2,4]triazin-7-yl)-5-cyano-3,4-dihydroxytetrahydrofuran-2-yl)(phenoxy)phosphoryl)-L-alaninate is the IUPAC name for it. The RMD powder is >98% pure. The best storage conditions for RMD are dark, dry, and at 0–4 °C for short-term storage (up to weeks) and at −20 °C for long-term storage (up to years). When RMD is properly stored, its shelf life is more than two years when dissolved in DMSO. In recent years, many countries have approved RMD for emergency treatment of COVID-19, especially when COVID-19 pandemics arise [18]. In India [19], Singapore [20], Japan [21], and the European Union, as well as in the United States and Australia, it has been approved for emergency use [22,23,24,25,26,27,28,29]. Starting in February 2021, the CHMP of the EMA has modified the indication for RMD to include those who do not require supplemental oxygen [30]. RMD has a molecular weight of 602.6 g/mol and is poorly soluble in water. It is given intravenously to adults and children under 40 kg at a dose of 200 mg once, then 100 mg once a day for a period of 5 to 10 days. Pharmacokinetic studies show that people with normal kidney function remove RMD and its active metabolite mostly (74%) through the kidneys. In comparison to the RMD parent, the active metabolite RMD triphosphate has a long plasma half-life (between 20 to 25 h) and is widespread throughout the body [31,32].

### 1.4. Research about RMD

**Figure 1:**
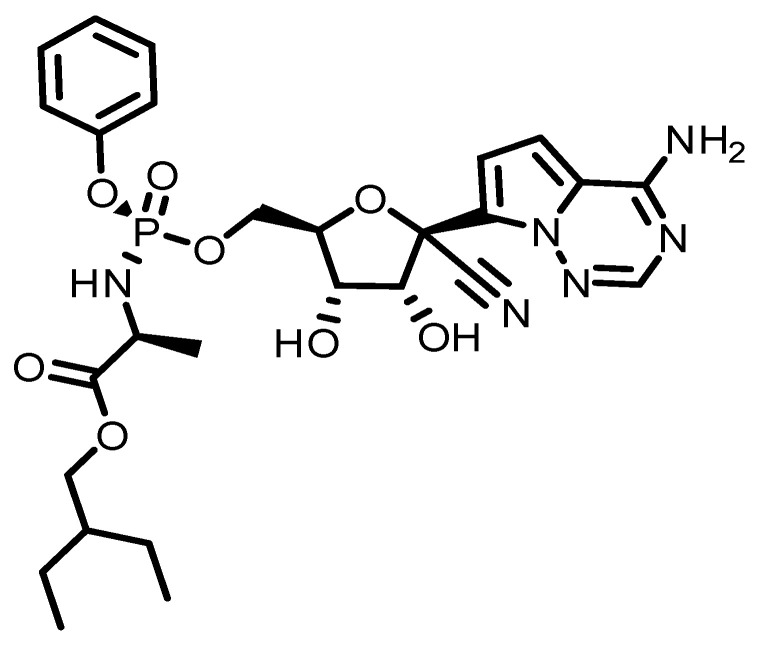
A description of the structure of RMD.

**Figure 2:**
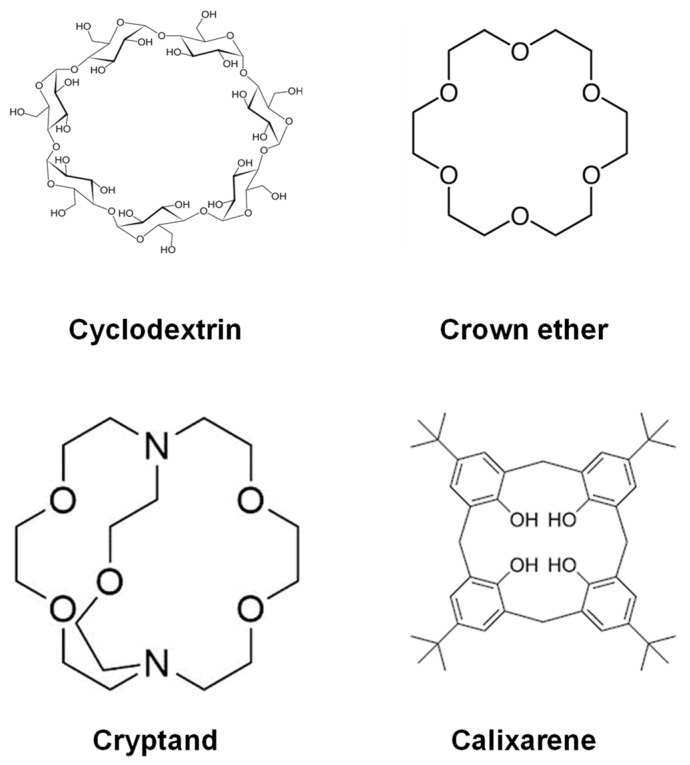
Various types of hosts.

This review has been compiled based on the results obtained from keyword (Remdesivir) searches in SciFinder and Google (details are presented in Supplementary Materials). RMD was developed by Gilead Sciences in 2009 to treat hepatitis C and RSV. However, it did not effectively treat such viruses [33,34]. Then it was processed and tested for treatment of the Ebola and Marburg viruses [30,34]. According to the Czech News Agency [35], this research was conducted under the supervision of Professor Tomas Cihlar. Scientists from Gilead Sciences and the CDC demonstrated the antiviral activity of RMD against several filoviruses, pneumoviruses, and paramyxoviruses in vitro [36]. Several government and academic institutions collaborated with Gilead Sciences on preclinical and clinical research [37,38,39,40]. However, RMD has also been investigated as a potential treatment for other viral infections, including coronaviruses such as SARS-CoV-2, the virus responsible for the COVID-19 pandemic [41]. RMD works by inhibiting the replication of the virus. It acts as a nucleotide analog, meaning it resembles the building blocks of viral RNA. When the virus attempts to replicate its genetic material, RMD is incorporated into the growing RNA chain, which ultimately leads to premature termination of the chain and prevents further viral replication. The drug gained significant attention and was granted emergency use authorization by the U.S. Food and Drug Administration (FDA) in May 2020 for the treatment of hospitalized COVID-19 patients. It was shown to shorten the recovery time in some patients and reduce the severity of the illness, although the clinical benefits were found to be modest. It is always a good idea to consult the latest scientific and medical resources or seek advice from healthcare professionals for the most up-to-date information. COVID-19 has a single-stranded RNA genome that requires a replication modifying domain to replicate. In its triphosphate form, RMD (Figure 1) acts as an analog to ATP, competing with RMD for uptake and hindering viral replication (Figure 2). In both in vitro and animal models, it has shown activity against severe acute respiratory syndrome coronavirus. The drug was originally intended as an Ebola-fighting agent [42,43].

### 1.5. Supramolecular Assembly with Hosts

The supramolecular assembly can be considered a different kind of macromolecule connected to a large structure by the host and guest interaction. Assemblies of supramolecular molecules are held together by interactions other than covalent bonds. For assembling supramolecular structures, hydrogen bonding is the main force [44]. The development of supramolecular structure is influenced by other forces, including solvent rearrangement, hydrophobic effect, and van der Waals interactions [45]. In the Netherlands, Bert Meijer of Eindhoven Technical University has developed several systems based on supramolecular assembly. The solubility, chemical reactivity, spectroscopic, and electrochemical properties of the guest molecules are all significantly impacted by the creation of the supramolecular assembly. The majority of these effects can be used in a variety of industries, such as the pharmaceutical sector [46,47], to increase the above-mentioned properties of medicines. These supramolecular structures can also be used in many disciplines of analytical chemistry, and biological systems, to delay the release of active chemicals from the pharmaceutical matrix, and as transporters of active substances [48,49].

The supramolecular assemblies in biology are almost similar to enzymes and the substrates they work with, with molecules as the guests that bond inside the cavities of hosts like CDs. The host molecule has a cavity or hole that can fit another molecule like a guest. The effort is comparable to trying to stuff a basketball into a peach basket. The figure above illustrates an example of host molecules (Figure 2). Each of them has an enclosed environment. It does not matter if it is just a ring in certain circumstances, it stops the guest from escaping [50]. However, in general, apart from CDs, some of the other systems like crown ethers, cryptands, and calixarenes are widely employed as host molecules or building blocks of supramolecular systems. One of the characteristic functions is ion selectivity [51,52]. Crown ether and calixarenes are generally used as host molecular materials to accommodate the guest molecule and are used for sensing as well as fuel production [53,54].

## 2. Cyclodextrin Chemistry

### 2.1. Introduction to Cyclodextrins

An enzymatic hydrolysis system forms cyclic polysaccharides like CDs from starch. In their native form, they are available in three variants: α-CD, β-CD, and γ-CD having six, seven, and eight glucopyranose units, respectively. CDs have truncated cone-shaped structures and the interiors of CDs are hydrophobic cavities; the exteriors are hydrophilic from the presence of hydroxyl groups. The three natural variants such as α-CD, β-CD, and γ-CD have internal cavities with diameters in the ranges of 4.7 to 5.3 Å mm, 6.0 to 6.5 Å mm, and 7.5 to 8.3 Å mm, respectively [55,56]. This material is ideal for packaging a variety of hydrophobic medicinal molecules inside the cavity. ICs are formed between the hydrophobic center and the hydrophobic medicinal molecules or biologically important molecules. Since CD has gained a lot of attention in the past few years, researchers have examined its potential for use in the pharmaceutical, food, and medical industries [57,58].

### 2.2. Formation of ICs through Host–Guest Interactions

**Figure 3:**
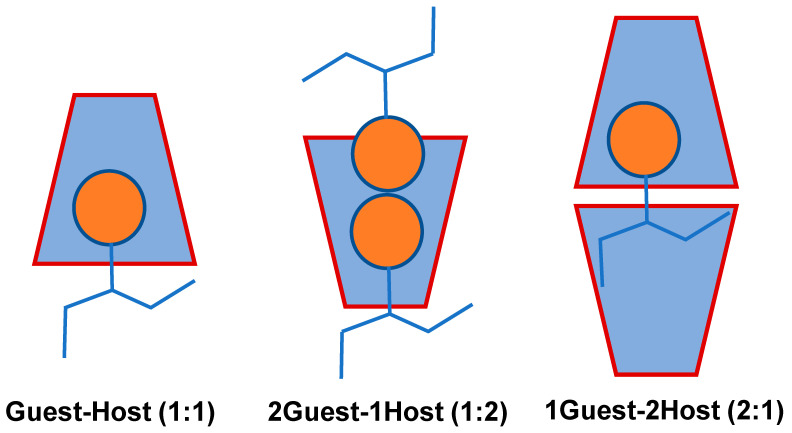
Possible Guest–Host stoichiometric ratio between guest and host (α, β, and γ-CDs) of the ICs [58].

Water molecules around the CD cavity have an energy structure that is not favored by the presence of water molecules (polar-to-non-polar interactions) and this can be spontaneously replaced by a guest molecule having a polarity lower than that of water molecules [59,60,61]. This formation of ICs is driven by the replacement of the high-enthalpy water molecules with the CD dissolved in water (Figure 3). Several CD moieties may participate in these ICs by entrapping one or more guest molecules, the most common being the 1:1 complexation [60,61]. In contrast, a more stable, crystalline molecule can undergo a more complexation [62,63], such as 2:1, 1:2, or even 2:2. The stability constant of the complex, K_a_ measures how quickly they establish equilibrium after being dissolved in aqueous environments [64,65].

### 2.3. Modification of Cyclodextrin

It is important to point out that two important factors influenced the chemical modification of CD: the nucleophilic character of the hydroxyl group and the behavior of the chemical reagent used. The low solubility of the native CD makes it unsuitable for pharmacological applications, despite its versatility in forming inclusion complexes with small molecules like organic and drug molecules, despite having an effective inner diameter. Creating water-soluble CDs is a primary goal of CD development. This resulted in several CD derivatives with better water solubility profiles than the original CDs. The following are the methods for preparing native CDs and their derivatives to overcome the disadvantages discussed earlier. Modified or derivatives of CDs can be produced by chemical or enzymatic reactions [66].

### 2.4. Derivatives of Cyclodextrin

The solubility of natural CDs and their complexes in water is quite limited, particularly for the β-CD. CD molecules have relatively strong bonds because of their crystal state [67]. The free hydroxyl groups in CDs come in three varieties, each with its specific function and reactivity. Each glucopyranose molecule has three free hydroxyl groups. Depending on the circumstances in which they are formed, such as pH, temperature, and reagents, CD’s secondary hydroxyls at the positions C-2, C-3, and C-6 as well as its primary hydroxyls at the positions, C-1, C-4, and C-5 respond differently. Various derivatives of CDs are produced by substituting the hydrogen and hydroxyl groups with different substituents like alkyl, hydroxyl alkyl, carboxylic acid, amino, thio, tosyl, glucosyl, and maltosyl groups. As a result, primary and secondary hydroxyl groups of the CDs can be modified to be carbonyl, carboxylic acid, benzyl, amino, thio, tosyl, and glucosyl.

Chemically changed CD derivatives are divided into two categories: (a) chemically modified CD derivatives, and (b) naturally enzymatically modified CD derivatives. These innovative chemically modified CD derivatives achieve the following goals: improving the fit and binding of CDs with their respective guests and obtaining insoluble, immobilized structures and polymers incorporating CDs, such as a chromatographic marker [68,69]. CD derivatives that are branched are formed with the aid of the pullulanase enzyme. Upon combining with a chemical reagent, the hydroxyls of β-CD form a heterogeneous product [57]. Several approaches can be used to describe and convey the extent of substitution. In CD molecules, the substitution degree indicates how many of the hydroxyl groups of the glucose moiety have been substituted. Thus, it could range between 1 and 3. As a result, the average DS is the average number of hydroxyl groups substituted in a glucose unit of CD. It might be between 0 and 3 [70].

An MDS is an essential parameter that is defined as the average number of substituents that can react completely with one glucopyranose repeat unit. When DS = 0, there is no substitution, or when DS = 1 to 3, there is oligomerization or polymerization due to the reaction of two or more substituents [71,72]. The hydroxypropyl derivatives of β and γ-CDs (HP-β-CDs and HP-γ-CDs), the RM-β-CDs, the SBE-γ-CDs, as well as branched CDs like Me-β-CDs are all products of great interest to the pharmaceutical industry [73].

### 2.5. Toxicological Importance of CDs

For their biomedical applications, CDs must possess some critical features, such as biocompatibility and biodegradation. The CD is a biocompatible pharmaceutical excipient and has been found in a wide spectrum of biological use [74]. Even though they have many applications, some critical remarks must be considered in their uses in in vivo performances. CDs are relatively stable materials that completely resist the degradation by human enzymes, and, in this regard, it is reported that after intravenous uptake of CD by humans, they are expelled intact via the kidney [75]. On the other hand, the CD can be degraded by bacterial and fungal enzymes (i.e., amylases), and hence, in the body, the CD is metabolized in the colon before being expelled [76]. Thus, CDs and guest molecules are involved in the normal biological pathway to be metabolized and expelled from the human body. CDs are generally non-toxic chemical materials that can potentially be used in different biological applications [77].

**Figure 4:**
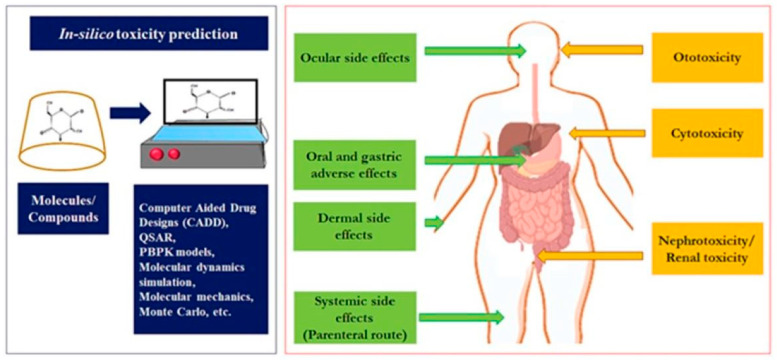
Adverse and toxicological effects of CDs. Reprinted with permission from Ref. [78]. Copyright 2024 Elsevier.

Toxicological studies are crucial in assessing the nature, characteristics, suitability, and safety of various drugs (such as anticancer and anti-inflammatory agents) and excipients. Given the widespread use of CDs across pharmaceutical, biomedical, and food industries, it is essential to evaluate their potential adverse reactions and toxic effects, and establish appropriate dosage requirements to ensure safety. CD-related toxicities can lead to serious side effects or disrupt normal physiological processes, which may occur when CDs are used in various delivery systems or nanocarriers. Figure 4 provides a summary of these toxic and adverse effects.

Although CDs and novel CD-based delivery systems and nanocarriers have been studied for their cytotoxic properties, there is a fundamental lack of dedicated toxicological data on CDs themselves that account for adverse reactions in the human body. Additionally, there is limited information on the toxicity of CDs resulting from acute or repeated exposure over extended periods. In CD-based nanocarriers loaded with drugs or other delivery systems, where the active compounds occupy the hollow cavity of CDs, cytotoxicity is often attributed to the increased permeability and uptake of the drugs, rather than the CDs themselves. However, in gene therapy, oligonucleotides can lead to an unwanted increase in immunogenicity due to enhanced permeation and solubility [79,80,81]. Similar to the increased toxicity of active compounds, CDs may also carry the potential for cumulative adverse effects over time. Therefore, further research is needed to establish the authentic and inherent toxicities of CDs in various pharmaceutical dosage forms.

## 3. Action on Supramolecular Complexes with Antiviral Drugs

CDs help in the containment of infection caused by enveloped viruses, such as coronaviruses and influenza viruses, by binding their envelopes to cellular receptors and merging them with the host cell membrane. For enveloped viruses to enter host cells, microdomains of cholesterol are required on viral envelopes and cell membranes. CDs reduce cholesterol’s presence in viral particles, causing their lipid rafts to rupture and structural damage to occur [82]. Cholesterol-binding agents such as digitonin, saponin, filipin, nystatin, and methyl-β-cyclodextrin (Me-β-CD) can rapidly remove cholesterol and disrupt lipid rafts. Unlike other cholesterol-binding agents that integrate into membranes, Me-β-CD is a strictly surface-acting agent. It selectively and quickly removes cholesterol from the plasma membrane while sparing other membrane lipids, making it a valuable tool for studying cholesterol depletion and lipid raft disassembly [83,84,85,86]. Moreover, CDs reduce the susceptibility of host cells to virus infection by removing cholesterol from their membranes. Through cholesterol depletion, Me-β-CD prevents influenza A and coronavirus infections [87,88]. It is possible to produce skin disinfection treatments by utilizing this feature of CDs. There are nasal sprays and throat sprays that can be produced to block the transmission of viruses through the respiratory system. Due to their biocompatibility, CD compositions can be used on skin and mucous membranes. There is an urgent need for effective treatments for COVID-19, which is spreading over the world rapidly. In response to this epidemic, companies are speeding up the development of medicines; however, formulation development is crucial for any potential drug. The HP-β-CD excipient may enhance the solubility and stability of pharmaceutical products including antivirals, monoclonal antibodies, and vaccine adjuvants. It is possible to contain infections with virucidal agents or modified CDs.

Oral antiviral therapies must have adequate solubility to ensure bioavailability and efficacy. Parenteral medicines have the advantage of rapid onset when administered to critically ill patients due to their solubility; medicines must be free of particles and buffered to physiological pH. Antiviral drugs can be improved in their solubility and bioavailability by utilizing CD drug delivery systems. Moreover, cyclodextrins that have been chemically changed are created by functionalizing their hydroxyl groups with a variety of substituents and combinations thereof. They mostly increase the solubility of medications that dissolve poorly in water [89]. HP-β-CD is found to be superior to other substituted β-CD in terms of improving the dissolving rate and solubility of the antiviral medicine, lopinavir (LPV). 2-hydroxypropylated (HP) derivatives are among the derivatives with the most widespread use, in particular HPβCD for their high safety and tolerability [90,91]. HP-β-CD is approved for use in oral and parenteral formulations due to its excellent safety profile even at relatively high doses, and is also listed on the FDA’s list of inactive pharmaceutical ingredients [92]. Drug delivery systems incorporating CD can address the formulation issues of antiviral medicines by improving solubility and bioavailability. To date, several articles have been published addressing the challenges resolved through the use of cyclodextrins (Table 2).

## 4. Modified Cyclodextrin with Virucidal Activity

Antiviral activity can be produced using modified CDs. Mercapto undecane sulfonic acid acts similarly to β-CD in its ability to inhibit viral replication, which is a broad-spectrum antiviral molecule that is rendered ineffective when diluted. A variety of different model animals and organisms are used in this regard, but mice are especially useful as they share mammalian features with humans and are affected by many of the same diseases [99]; the modified CDs are virucidal against a wide range of viruses in vitro and in vivo at micromolar concentrations [100]. This is because CDs can be modified in a variety of ways to produce antiviral activity without harming the body.

Coronaviruses as well as influenza viruses infect cells by binding to the receptors on cellular membranes and fusing with them. For enveloped viruses to successfully enter host cells, cholesterol must be present in the microdomains of the viral envelope and the cell membrane. CDs sequester lipids from viral particles, resulting in structural deformation of the viral envelope due to disruption of the lipid rafts [82]. In addition to depleting cholesterol from host cell membranes, CDs can also reduce their susceptibility to viral infection. A recent study demonstrated that Me-β-CD reduces coronavirus infection and influenza A virus infectivity through the depletion of cholesterol [87,88]. It may be possible to harness this property of CDs for developing solutions and also for skin disinfection. Additionally, aerosol sprays can be developed as a preventive measure to ensure that viral transmission does not occur through the respiratory system. The biocompatibility of CD formulations with skin and mucous membranes makes them attractive.

**Figure 5:**
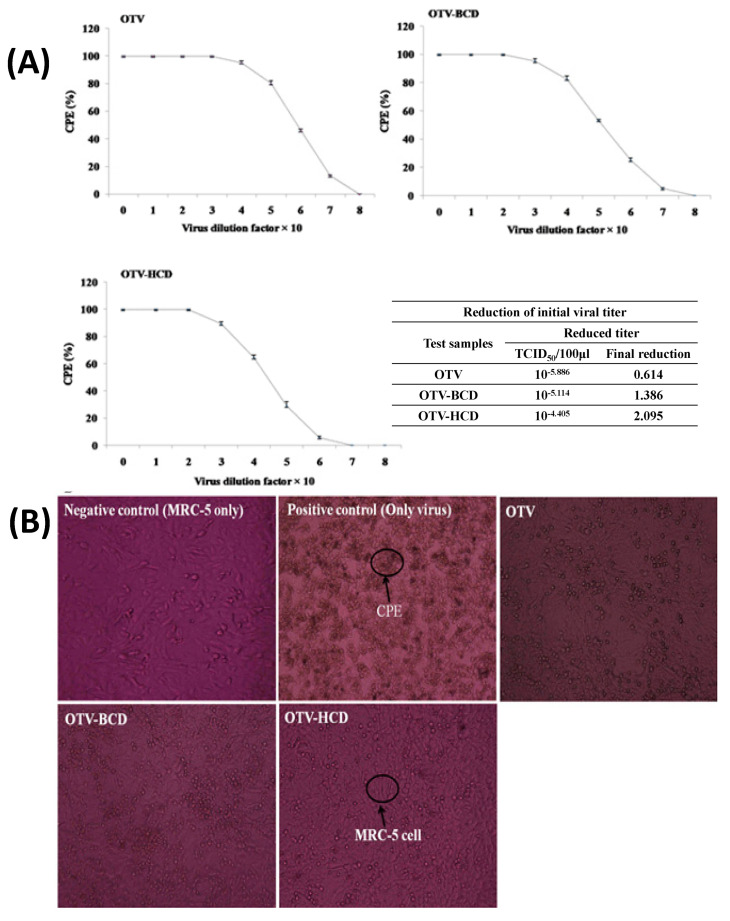
CD inhibited the cytopathic effect (CPE) in lung epithelial cells (MRC−5) and reduction of virus titer (**A**), and cytopathic effect (CPE) in lung epithelial cell (MRC−5) with OTV and ICs (**B**). Reprinted with permission from Ref. from [96], Copyright (2024) Elsevier.

The drug molecule OTV, along with its ICs formed with both native and modified CDs, has been tested for efficacy against coronavirus. The results demonstrated a significant improvement in antiviral activity without any observed toxicity [96,97]. Specifically, the gradual release of OTV from the ICs is found to enhance virus inactivation. When MRC-5 cells are introduced, a decrease in syncytium formation is observed in the ICs, suggesting improved inactivation (Figure 5). This marks the first report of virus inactivation facilitated by water-soluble ICs. The control cells displayed normal morphology. Moreover, OTV showed a lower total protein expression (TPE) compared to OTV, indicating potentially superior performance. Consequently, the research successfully achieved its objectives.

## 5. Interaction of RMD with Cyclodextrins

**Figure 6:**
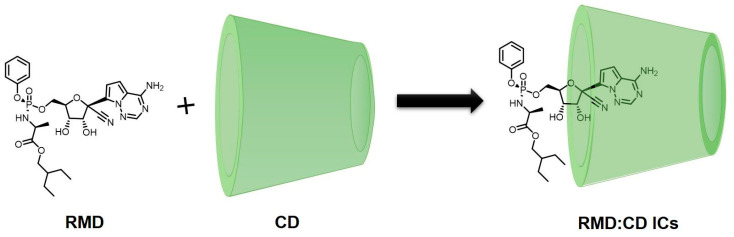
Interaction of RMD with CDs (proposed structure).

Table 3 has been consolidated to include information on the formation of ICs of RMD within the cavities of both native and modified CDs, as determined by various experimental and computational methods, and the outputs revealing RMD exhibits strong interaction with native and modified β-CD [100,101,102,103,104,105,106,107]. According to the information discussed above regarding the CD’s ability to bind organic drug molecules, it can either fully or partially bind the drug molecule depending upon its size. In some cases, the functional group of the drug molecule is incorporated into the cavity [108,109]. Occasionally, another part of the drug may be inserted into the cavity of the CDs [110,111]. Molecular dynamics, as well as molecular mechanics, can be applied to the explanation of the penetration of the drug into the cavity of the CDs [112]. An interaction between drugs and CDs has also been proposed using the computational approach. Taking this into account, the interaction of RMD with CDs has been simplified as follows (Figure 6).

### 5.1. RMD Interactions with β-CD by the Experimental Method

RMD has limited water solubility and requires an excipient that is suitable for solubilization, such as native or modified CD at pH 2. To achieve this, Bianka Várnai used a final formulation that includes the randomly replaced SBE-β-CD as a complexing agent [100,113]. Several CD derivatives were used to investigate the molecular interactions in RMD:SBE-β-CD ICs. Due to the presence of permanent negative charges on the aminopyrrolotriazine moiety in SBE-β-CD, the protonation state of the RMD moiety plays an important role in the formation of CD-RMD ICs. A pK_a_ of 3.56 was determined using a UV-pH titration study on RMD, due to its positive charge at pH 2.0. γ- and β-CD derivatives were analyzed using NMR to determine their stability, stoichiometries, and structures. The stability constants were established by nonlinear curve fitting at pH 2.0 based on ^1^H NMR titrations, while the stoichiometry was determined by Job’s method. There was a significant difference in the stability of β-CD complexes compared to γ-CD complexes.

RMD-per-6-SBE-β-CD demonstrated high stability values during sulfobutylation (log K = 4.35 for RMD-per-6-SBE-β-CD). In the case of γ-CDs, it is the phenoxy moiety that overtakes and promotes RMD inclusion, while the ethylbutyl moiety is crucial for the integration of complexation into the cavity of the β-CD. The formulation and characterization of RMD and CD derivatives with specific stabilities can be an aid in the formulation and manufacture of RMD, in addition to the analysis of RMD:β-CD ICs. Hence, the aforementioned findings also show that the charge states of the host and the guest may significantly influence the ICs of RMD. Also, it may pave the way for the use of CDs in other areas as well, as the formulation, preparative scale purification, and chiral analysis of RMD all present problems to the analytical community.

SBECD, with an average degree of substitution of 6.5 sulfobutylether groups (SBE = 6.5), serves as a solubility enhancer for RMD and is a key component in Veklury. Veklury is approved by the FDA for treating COVID-19 in patients aged 12 and older, weighing over 40 kg, who require hospitalization [105]. However, due to the renal filtration-based clearance of SBECD, there is concern about its potential accumulation in patients with impaired renal function receiving Veklury. To address this issue, a highly specific, accurate, and precise LC-MS/MS method was developed and validated for determining SBECD concentrations in human plasma. This method is now being utilized to monitor SBECD levels in pharmacokinetic studies involving pediatric patients.

### 5.2. RMD Interactions with SBE-β-CD by Molecular Dynamics Simulations

**Figure 7:**
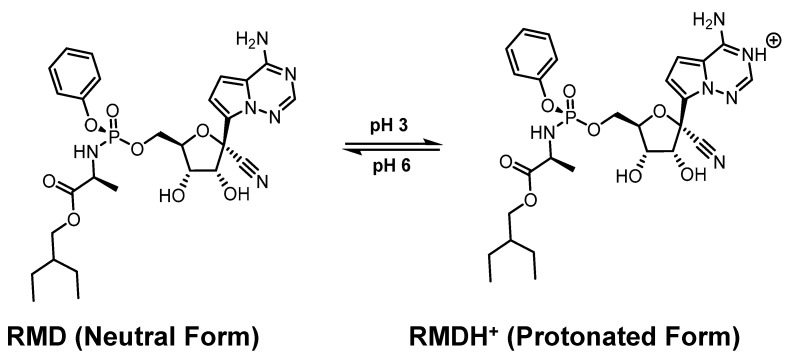
The uncharged (neutral) and charged (protonated) form of RMD. Reprinted from Ref. [101].

Multiple isothermal titration calorimetric (ITC) studies conducted at two pH levels by Rebecca Garcia-Fandino et al. revealed that RMD and SBE-β-CD interact with each other [101]. With the help of biased molecular dynamics (MD) simulations using neutral and protonated forms of RMD, multiple isothermal titration calorimetry (ITC) experiments at two different pH levels, three different structures for SBE-β-CD with substituted groups of 5, 6, and 7, and biased MD simulations, this part of work aims to shed some light on this issue. As a result of biased MD simulations, RMD exists at pH 6 and pH 3 as a neutral and protonated form, respectively (Figure 7). Here are how those results were interpreted. In a worldwide analysis of the calorimetry results, ICs of fairly high affinity are formed at pH 3 with a stability constant of 104. A large number of structures are insufficient to fully encapsulate RMD because an additional parameter must be included in the fitting model to account for the amount of active CD. At neutral pH, the ITC measurements provided no strong signal. This may be due to weak interactions like hydrogen bonding, but the relevant isotherms could not be determined conclusively. As a consequence of CD dilution, the signals obtained from the interaction with the RMD molecule were comparable in both pH settings. It is still necessary to investigate the source of this signal, but considering its importance, the CD should be compared with other drugs or RMD. In contrast to ITC analysis, which provides global thermodynamic characteristics for the full distribution of CD structures, RMD encapsulation is not determined by the frequency and position of SBE substitutions.

**Figure 8:**
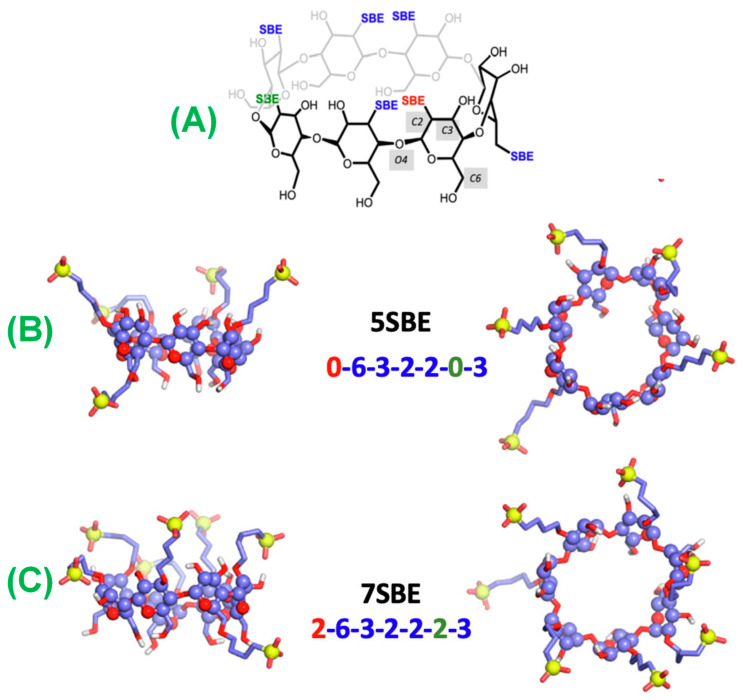
Structures of (**A**) SBE-β-CD, (**B**) 5SBE, and (**C**) 7SBE after 500 ns of MD simulations show the water molecules at less than 3 Å from any atom of the CDs. Carbon is represented by violet, oxygen by red, hydrogen by white, and sulfur by yellow. Sulfur atoms and glucopyranoside rings are represented by spheres, while oxygen and carbon atoms are represented by sticks. Reprinted from Ref. [101].

To investigate this further, MD simulations were performed with three different structures with SBE substitutions of A, B, and C (Figure 8). Simulation results indicate that the RMD molecule interacts with the CD primarily on its secondary side and preferentially in its up position. There is a high energy barrier on all structures that may impede the entry of RMDs via the CD’s primary side. Moreover, according to the simulations, the RMD binding is much stronger when it is protonated (at the lower pH values) than when it is not protonated (at neutral pH values). The association constants determined from the PMF profiles indicate that the structure with the lowest number of SBE substitutions has a weaker binding. This agrees with the ITC conclusion that the affinity depends primarily on the number and placement of CD-containing structures. It would be helpful to confirm our findings by examining other CD structures with fewer or more substitutions, as well as with complementary experimental and computational techniques. It may be advantageous to conduct complementary studies using many CD molecules in the same simulation box to account for self-aggregation.

In general, the results suggested that pharmaceutical formulations that concentrate the most active fractions could improve their efficacy. Thus, future toxicity concerns may be reduced, and treatments for COVID-19 may be able to be given at lower pharmacological doses.

### 5.3. RMD Interactions with SBE-β-CD by the Experimental Method

Through experimental and molecular modeling studies, the interaction between RMD and SBE-β-CD has been explored thoroughly [102]. Powder XRD and DSC studies are carried out on lyophilized and electrospun forms of goods to determine whether the crystalline structure has been completely amorphized. Raman chemical mapping techniques and SEM-EDS techniques demonstrated the homogenous distribution and molecular dispersion of RMD in the carbohydrate matrix.

To discover SBE-β-CD ICs, the RMD examined aqueous solutions with ROESY spectroscopic analysis. In an acidic solution, RMD remains inside the cavity of SBE-β-CD despite the presence of its ethyl-butyl side chain. However, the positively charged heterocyclic moiety does not incorporate into SBE-β-CD. Together with the inclusion phenomena, electrostatic and hydrogen bond-related interactions also contribute to the development of stable ICs. Stable integrated circuits require hydrogen bonds as well as electrostatic interactions in addition to inclusion processes. Under neutral pH conditions, computer docking tests provided insight into the molar stoichiometry of the ICs and the insertion of RMD into the SBE-β-CD cavity. Several types of molecular interactions have been observed between RMD and polyanionic CD derivatives of SBE-β-CD polyanionic CD [114].

Computational investigations aligned with experimentally determined phase solubilization, providing a clear explanation of the macroscopic clathration mechanism between RMD and SBE-β-CD through microscopic molecular calculations. Using accurate quantum mechanics (QM)-based pK_a_ predictions, a novel approach combining QM, molecular dynamics (MD), and molecular docking was developed to account for the observed macroscopic behavior of RMD in SBE-β-CD. This research highlights the effectiveness of integrating experimental data with mechanism-based computational methods for studying complex drug–excipient interactions [104].

### 5.4. RMD Interactions with CDs for Enhanced Solubility

A comprehensive study [103] was conducted to assess whether the protonation state of the drug molecule and the number of substitutions within the cavity of CD influence the formation of aggregates. The findings indicate two opposing effects as the number of substitutions in SBE-β-CDs increases: while it strengthens the interaction with the drug, particularly in its protonated form, it slightly decreases the ability to prevent the self-aggregation of RMD. This suggests that a lower concentration of excipient might be required to solubilize RMD if the distribution favors a higher degree of substitution. Regarding solubilization, the phase solubility of RMD showed a significant increase from 1.7 mmol/L at 25 °C to 60 mmol/L at 37 °C in pH 1.5 solutions, as the concentration of SBE-β-CD rose from 0 to 185 mM [104].

### 5.5. CDs for Infection Containment

A virus’s ability to bind to cellular receptors, such as coronaviruses and influenza viruses, and fuse with the host’s cell membrane is the basis for infection. For enveloped viruses to enter the host cell, it appears that cholesterol present in the microdomains of the viral envelope and the cell membrane is necessary. CDs can sequester cholesterol from viral particles and disrupt the lipid raft, resulting in structural deformation of the viral envelope [77]. As a result, CDs lower cholesterol levels in the membranes of host cells, lessening their vulnerability to viral infections. Using Me-β-CD, cholesterol depletion has been shown to decrease viral infectivity in coronaviruses and influenza A viruses [87,88]. There is a property of CDs that makes them useful in making skin disinfection treatments. As an additional measure, viral transmission can be inhibited via the respiratory pathway through nasal and throat spray. The biocompatibility of CD formulations with skin and mucous membranes is one of their advantages.

### 5.6. Future Perspectives

The host–guest assembly of RMD with CDs holds immense potential for addressing the challenges associated with coronavirus resistance. However, further research is necessary to optimize this approach and expand its application. Firstly, investigations should focus on elucidating the mechanistic details of the ICs between CDs and RMD. This understanding will facilitate the rational design of cyclodextrins with enhanced encapsulation efficiency and antiviral activity. Additionally, the development of novel CD derivatives with improved biocompatibility and targeted drug delivery capabilities is an avenue that warrants exploration. Tailoring the physicochemical properties of CDs can potentially enhance their interactions with RMD and enable site-specific drug release, thereby maximizing therapeutic efficacy and minimizing off-target effects. Furthermore, in vivo studies and clinical trials are essential to validate the potential of CD-based formulations of RMD in real-world scenarios. Rigorous evaluation of their safety, pharmacokinetics, and efficacy against drug-resistant coronavirus strains will be crucial for their successful translation into clinical practice.

## 6. Conclusions and Remarks

The concept of complexation phenomena with CD cavities has reached a significant level by providing beneficial modifications to the physicochemical properties of guest molecules. By the ICs, the solubility of the insoluble compound/drug is much improved, the stability of the guest-like currently used drugs that can resist the oxidation/UV radiation/heat is improved, the volatility and sublimation nature of the molecules/drugs are much more controlled, the unfriendly smells and essences are screened off, and drugs are released in a controlled manner. Many industries, including pharmaceuticals, utilize CDs as useful additions. CD has so far played a significant role in pharmaceutical and biomedical applications because it is biocompatible, biodegradable, and has a well-established safety profile in clinical use for humans. An inclusion complex is formed when CD binds to a variety of medicinal compounds. It is for this reason CD has so many benefits. One of the advantages and benefits of complexing CD with RMD is that the drug can be used effectively after it has been made. Moreover, it can serve as a capping agent while it is packaged and also as an intake agent when patients suffering from COVID-19 need it. An RMD can be free from contaminating or polluting factors such as heat, humidity, or chemical substances such as solvents or impurities due to the formation of a CD complex when using native as well as modified CDs. The design of new native, as well as the CD derivatives with customized stabilities, is now possible for the investigation of RMD:CD ICs. This will help with the formulation, manufacture, and even analytical characterization of RMD. The host–guest assembly of RMD with CDs represents a promising approach to overcoming coronavirus resistance. With its ability to enhance the solubility, stability, and bioavailability of RMD, this strategy holds great potential for optimizing antiviral therapy. However, continued research efforts are needed to fully exploit the benefits of this host–guest assembly and pave the way for effective clinical implementation in the future.

## Figures and Tables

**Table 1:** Antiviral drugs with their action and their efficiency against COVID-19. (This review has been compiled based on the results obtained from keyword searches in SciFinder and Google, details are presented in Supplementary Materials).

S. No	Drugs	Mechanism of Action	Usage	Efficacy	References
1	Remdesivir (RMD)	It is a nucleotide analog prodrug that inhibits the viral RNA-dependent RNA polymerase (RdRp), a crucial enzyme for SARS-CoV-2 replication.	Approved by the FDA for emergency use in hospitalized COVID-19 patients.	Clinical trials (e.g., ACTT-1) have shown that RMD reduces the recovery time in hospitalized patients.	[6,7,8]
2	Molnupiravir (MNP)	MNP is a prodrug of the nucleoside analog N4-hydroxycytidine, which introduces copying errors during viral RNA replication.	Administered orally and primarily used in outpatient settings for patients with mild to moderate COVID-19 who are at high risk of progression to severe disease.	MNP has been shown to reduce hospitalization and death rates in non-hospitalized patients when given early in the course of infection.	[7,9,10,11]
3	Paxlovid (Nirmatrelvir + Ritonavir)—PXLD	PXLD is a combination of nirmatrelvir, a 3CL protease inhibitor that prevents viral polyprotein cleavage, and ritonavir, a pharmacokinetic enhancer that increases nirmatrelvir’s half-life by inhibiting its metabolism. Blocking the 3CL protease (Mpro) disrupts viral replication at an early stage.	This oral PXLD is prescribed for high-risk, non-hospitalized patients with mild to moderate COVID-19.	Clinical trials have shown Paxlovid reduces the risk of hospitalization or death by nearly 89% in high-risk patients when administered early.	[7,9,12,13]
4	Favipiravir (Avigan)—FVP	FVP is a purine nucleoside analog that inhibits RdRp, similar to RMD, by incorporating itself into viral RNA, leading to chain termination.	FVP has been used in some countries (e.g., Japan, India, Russia) for treating mild to moderate COVID-19	Clinical efficacy data for FVP in COVID-19 are mixed.	[6]
5	Baricitinib (Olumiant)—BRC	It inhibits the Janus kinase (JAK-STAT) pathway, reducing the inflammatory response associated with severe COVID-19, and also potentially interferes with the virus’ entry into cells by inhibiting AP2-associated protein kinase 1 (AAK1).	BRC is used in combination with RMD for treating hospitalized COVID-19 patients.	BRC combined with RMD reduced recovery time and improved clinical outcomes compared to RMD alone.	[6]

**Table 2:** List of antiviral drugs currently being tested or in development to treat COVID-19.

S. No	Antiviral Drugs	CDs Used	Regulatory Status	Challenges Faced	Reference
1.	Remdesivir	SBE-β-CD	Compassionate use/clinical trials	Limited Solubility	[93]
2.	Lopinavir + Ritonavir	HP-β-CD	Approved anti-HIV drug	Limited Solubility	[94]
3.	Oseltamivir	β-CD	Approved anti-influenza drug	Bitter Taste	[95]
4.	Oseltamivir	β-CD	Article published	Human coronavirus	[96]
5	Oseltamivir	HP-β-CD	Article published	Human coronavirus	[96]
6.	Oseltamivir	M-β-CD	Article published	Human coronavirus	[97]
7.	Oseltamivir	S-β-CD	Article published	Human coronavirus	[98]

**Table 3:** Interaction of RMD with native and modified CDs.

S. No	Antiviral Drug	CDs Used	Method of Analysis	Outputs	References
1.	RMD	β-CD	Experimental method	Enhanced solubility	[100]
2.	RMD	SBE-β-CD	Molecular dynamics simulations	Confirmations of ICs in non-protonated and protonated form	[101]
3.	RMD	SBE-β-CD	Experimental method	XRD, DSC, and ROESY confirm the ICs	[102]
4	RMD	SBE-β-CD	Molecular dynamics simulations	Enhanced solubility	[103]
5.	RMD	SBE-β-CD	Experimental and docking method	Enhanced solubility	[104]
6	RMD	SBE-β-CD	Flow cytometry	Veklury^®^ formulations in meta-analyses of clinical trials	[105]
7	RMD	SBE-β-CD Sodium	Experimental method	Pharmacokinetics study for pediatric patients	[106]
8	RMD	HP-β-CD	Experimental method	Cellular uptake and cytotoxicity	[107]

## Data Availability

No new data were created or analyzed in this study. Data sharing does not apply to this article.

## Supplementary Materials

The following supporting information can be downloaded at: https://www.mdpi.com/article/10.3390/molecules29194782/s1, The methodology of the literature results which was obtained from keyword searches in SciFinder and Google.

## Abbreviations

RMD: Remdesivir (RMD); Cyclodextrin (CD); Cyclodextrins (CDs); Inclusion complex (IC); Inclusion complexes (ICs); β-Cyclodextrin (β-CD); Hydroxypropyl-β-Cyclodextrin (HP-β-CD); Sulfobutylether-β-Cyclodextrin (SBE-β-CD); Randomly methylated β-CDs (RM-β-CDs); Sulfobutylethylether salt of γ-CDs (SBE-γ-CDs); Hydroxyethyl-β-cyclodextrin (HE-β-CD); Heptakis (2,6-dimethyl)-β-cyclodextrin (DIME-β-CD); Heptakis (2, 3, 6-trimethyl)-β-cyclodextrin (TRIME-β-CD); Maltosyl-β-CDs (M-β-CDs); Methylated-β-CD (Me-β-CD); Acute Kidney Disease (AKI); End Stage Kidney Disease (ESKD); Human immunodeficiency virus (HIV); Zika virus (ZIKV); AP2-associated protein kinase 1 (AAK1); Degree of substitution (DS); Molar degree of substitution (MDS); Food and Drug Administration (FDA); Committee for Medicinal Products for Human Use (CHMP); Respiratory syncytial virus (RSV); Centers for Disease Control and Prevention (CDC); European Medicines Agency (EMA); Adenosine triphosphate (ATP); Isothermal titration calorimetry (ITC); X-ray diffraction (XRD); Differential scanning calorimetry (DSC); Energy dispersive X-ray spectroscopy (EDS); Scanning electron microscopy (SEM); Nuclear Magnetic Resonance (NMR); Proton Nuclear Magnetic Resonance (^1^H NMR); Rotating Frame Overhauser Enhancement Spectroscopy (ROESY); Molecular dynamics (MD); Ultraviolet (UV); Dimethyl sulfoxide (DMSO); nanosecond (ns); Stability constant (K_a_).

## References

[1] DeClercq E. (2002). Strategies in the design of antiviral drugs. Nat. Rev. Drug Discov..

[2] McCormack S., Ramjee G., Kamali A. (2010). PRO2000 vaginal gel for prevention of HIV-1 infection (microbicides development programme 301): Phase 3, randomised, double-blind, parallel-group trial. Lancet.

[3] Pirrone V., Wigdahl B., Krebs F.C. (2011). The rise and fall of polyanionic inhibitors of the human immunodeficiency virus type 1. Antivir. Res..

[4] Van Damme L.R., Govinden R.F.M., Mirembe F.M.F. (2008). Lack of effectiveness of cellulose sulfate gel for the prevention of vaginal HIV transmission. N. Engl. J. Med..

[5] Wieslaw M.K. (2011). Antiviral Drugs: From Basic Discovery through Clinical Trials.

[6] Laila R., Sunny O.A., Amanuel G.A., Mickael E., Aliyu Tijani J., Andrzej F., Robert F., Rangarirai M., Leander M., Kawthar M. (2022). Oral antiviral treat-ments for COVID-19: Opportunities and challenges. Pharm. Rep..

[7] Daisy Y., Bingfang Y. (2023). Viral target and metabolism-based rationale for combined use of recently authorized small molecule COVID-19 medicines: Molnupiravir, nirmatrelvir, and remdesivir. Fund. Clin. Pharmacol..

[8] Faez I., Tongzhou K., Haider A., Dakun L. (2021). Remdesivir Strongly Binds to RNA-Dependent RNA Polymerase, Membrane Protein, and Main Protease of SARS-CoV-2: Indi-cation From Molecular Modeling and Simulations. Front. Pharmacol..

[9] Parums D.V. (2022). Editorial: Current Status of Oral Antiviral Drug Treatments for SARS-CoV-2 Infection in Non-Hospitalized Patients. Med. Sci. Monit..

[10] Jayk Bernal A., Gomes da Silva M.M., Musungaie D.B., Kovalchuk E., Gonzalez A., Delos Reyes V. (2022). Molnupiravir for Oral Treatment of COVID-19 in Nonhospitalized Patients. N. Engl. J. Med..

[11] Mahase E. (2021). COVID-19: UK becomes first country to authorise antiviral molnupiravir. BMJ.

[12] Ledford H. (2021). COVID antiviral pills: What scientists still want to know. Nature.

[13] Owen D.R., Allerton C.M.N., Anderson A.S., Aschenbrenner L., Avery M., Berritt S. (2021). An oral SARS-CoV-2 Mpro inhibitor clinical candidate for the treatment of COVID-19. Science.

[14] Hannah A. (2023). Blair, Remdesivir: A Review in COVID-19. Drugs.

[15] Michael K.L., César G.A., Jason K.P., Silvia C., Egor P.T., Lisa G., Ayan C., Punya S.-R., Payel C., Laura K.M. (2020). Remdesivir targets a structurally analogous region of the Ebola virus and SARS-CoV-2 polymerases. Proc. Natl. Acad. Sci. USA.

[16] Chun B.K., Clarke M.O., Doerffler E., Hui H.C., Jordan R., Mackman R.L., Parrish J.P., Ray A.S., Siegel D. (2016). Methods for Treating Filoviridae Virus Infections. U.S. Patent.

[17] Clarke M.O., Jordan R., Mackman R.L., Ray A.S., Siegel D. (2017). Preparation of Amino Acid-Containing Nucleosides for Treating Flaviviridae Virus Infections.

[18] (2020). U.S. Food and Drug Administration Approves Gilead’s Antiviral Veklury (Remdesivir) for Treatment of COVID-19 (Press Release).

[19] Reuters (2020). India Approves Emergency Use of Remdesivir to Treat COVID-19 Patients.

[20] Reuters (2020). Singapore Approves Remdesivir Drug for Emergency COVID-19 Treatment.

[21] Pharmaceutical-Technology (2020). Japanese Regulator Approves Gilead’s Remdesivir to Treat COVID-19.

[22] European Medicines Agency (EMA) (2020). Veklury EPAR.

[23] Gilead Sciences (2020). Gilead Announces Approval of Veklury (remdesivir) in Japan for Patients with Severe COVID-19 (Press Release).

[24] U.S. Food and Drug Administration (FDA) (2020). Remdesivir EUA Letter of Authorization.

[25] U.S. Food and Drug Administration (FDA) (2020). Coronavirus (COVID-19) Update: FDA Issues Emergency Use Authorization for Potential COVID-19 Treatment (Press Release).

[26] The Asahi Shimbun (2020). Japan Approves Remdesivir for COVID-19 Despite Uncertainties.

[27] Therapeutic Goods Administration (TGA) (2020). Australia’s First COVID Treatment Approved.

[28] U.S. Food and Drug Administration (FDA) (2020). Veklury: FDA-Approved Drugs.

[29] U.S. Food and Drug Administration (FDA) (2019). Veklury: Summary Review.

[30] European Medicines Agency (EMA) (2021). EMA Starts Evaluating Use of Veklury in COVID-19 Patients Not Requiring Supplemental Oxygen (Press Release).

[31] Warren T.K., Jordan R., Lo M.K., Ray A.S., Mackman R.L., Soloveva V., Siegel D., Perron M., Bannister R., Hui H.C. (2016). Therapeutic efficacy of the small molecule GS-5734 against Ebola virus in rhesus monkeys. Nature.

[32] Gilead R. (2020). Summary on Compassionate Use.

[33] Stephens B. (2020). The Story of Remdesivir.

[34] (2020). Final Report Confirms Remdesivir Benefits for COVID-19.

[35] Czech News Agency (2020). Did Czech Scientists Create the Cure for Coronavirus?.

[36] Lo M.K., Jordan R., Arvey A., Sudhamsu J., Shrivastava-Ranjan P., Hotard A.L. (2017). GS-5734 and its parent nucleoside analog inhibit Filo-, Pneumo-, and Paramyxoviruses. Sci. Rep..

[37] Silverman E. (2020). U.S. Government Contributed Research to a Gilead Remdesivir Patent—But Didn’t Get Credit.

[38] Ardizzone K. (2020). Role of the Federal Government in the Development of Remdesivir.

[39] Savannah K. (2020). Investigational Compound Remdesivir, Developed by UAB and NIH Researchers, Being Used for Treatment of Novel Coronavirus.

[40] Eastman R.T., Roth J.S., Brimacombe K.R., Simeonov A., Shen M., Patnaik S., Hall M.D. (2020). Remdesivir: A Review of Its Discovery and Development Leading to Emergency Use Authorization for Treatment of COVID-19. ACS Cent. Sci..

[41] Sheikholeslami S.M., Jahanbani A., Shao Z. (2021). On the molecular structure of Remdesivir for the treatment of COVID-19. Comp. Meth Biomech. Biomed. Eng..

[42] Sheahan T.P., Sims A.C., Graham R.L., Menachery V.D., Gralinski L.E., Case J.B. (2017). Broad-spectrum antiviral GS-5734 inhibits both epidemic and zoonotic coronaviruses. Sci. Trans. Med..

[43] Wang M., Cao R., Zhang L., Yang X., Liu J., Xu M., Shi Z., Hu Z., Zhong W., Xiao G. (2020). Remdesivir and chloroquine effectively inhibit the recently emerged novel coronavirus (2019-nCoV) in vitro. Cell Res..

[44] Szejtli J., Osa T. (1996). Comprehensive Supramolecular Chemistry.

[45] Ali Aboel D. (2014). Rapid analysis of drug binding to β-cyclodextrin: Part II substituents effect on physicochemical and co-conformational stability of drug/cyclodextrin complex. RSC Adv..

[46] Diez M.A., Pena I.M.A., Garera M.C.M., Gil D.B., Canada F.C. (2007). Fluorimetric Determination of Sulphaguanidine and Sulphamethoxazole by Host-Guest Complexation in β-Cyclodextrin and Partial Least Squares Calibration. J. Flu..

[47] Radi A.-E.M., Eissa S.H. (2011). Voltametric and spectrophotometric studies on the inclusion complex of glipizide with β-CD. Eur. J. Anal. Chem..

[48] Cusola O., Tabary N., Belgacem M.N., Bras J. (2013). Cyclodextrin functionalization of several cellulosic substrates for prolonged release of antibacterial agents. J. Appl. Pol. Sci..

[49] Linare M., Bertorello M.M., Longhi M. (2000). Preparation and characterization of solid complexes of Naphtoquinone and Hydroxypropyl-b-cyclodextrin. Molecules.

[50] Kyeong R.R., Ji W.H. (2021). Chemical Interface Damping of Silver-coated Gold Nanorods Using Supramolecular Host–Guest Chemistry. Bull. Korean Chem. Soc..

[51] Sota T., Tomoyuki U., Mayuko K., Motoki K., Daisuke S., Satoru M., Manabu A., Takeharu H., Takayuki E., Fuminori M. (2020). Conformation of K^+^ (Crown Ether) Complexes Revealed by Ion Mobility–Mass Spectrometry and Ultraviolet Spectroscopy. J. Phys. Chem. A.

[52] Paul M., Scott J.D., Martin J.P. (2016). Transition Metal Complexes of Calix[4]arene: Theoretical Investigations into Small Guest Binding within the Host Cavity. J. Phys. Chem. A.

[53] Ju B.C., Jae S.H., Cheal K. (2021). Crown-Ether Type Chemosensor for the Determination of Fe3+/2+ by a Colorimetric Method. Bull. Korean Chem. Soc..

[54] Jumina I., Amalina S., Triono Y.S., Kurniawan Y.P., Keisuke O., Bohari M.Y. (2021). Preliminary Investigation of Organocatalyst Activity Based on C-Arylcalix[4]-2-Methylresorcinarene Sulfonic Acid Materials for Biodiesel Production. Bull. Korean Chem. Soc..

[55] Del Valle E.M. (2004). Cyclodextrins and their uses: A review. Process Biochem..

[56] Singh M., Sharma R., Banerjee U. (2002). Biotechnological applications of cyclodextrins. Biotechnol. Adv..

[57] Antía G.P., Maria C., Paula G.O., Juan C.M., Miguel A.P., Jesus S.G. (2021). Main Applications of Cyclodextrins in the Food Industry as the Compounds of Choice to Form Host–Guest Complexes. Int. J. Mol. Sci..

[58] Shery J., Anroop B.N. (2018). Cyclodextrin complexes: Perspective from drug delivery and formulation. Drug Dev. Res..

[59] Szente L., Szejtli J. (1999). Highly soluble cyclodextrin derivatives: Chemistry, properties and trends in development. Adv. Drug Deli Rev..

[60] Deluzio T.G.B., Penev K.I., Mequanint K. (2014). Cyclodextrin inclusion complexes as potential oxygen delivery vehicles in tissue engineering. J. Bio. Tissue Eng..

[61] Frank D.W., Gray J.E., Weaver R.N. (1976). Cyclodextrin nephrosis in the rat. Am. J. Pathol..

[62] Frömming K.-H., Szejtli J., Frömming K.-H., Szejtli J. (1994). Pharmacokinetics and toxicology of cyclodextrins. Cyclodextrins in Pharmacy.

[63] Szente L., Szejtli J. (2004). Cyclodextrins as food ingredients. Trends Food Sci. Technol..

[64] Loftsson T., Brewster M.E. (2010). Pharmaceutical applications of cyclodextrins: Basic science and product development. J. Pharm. Pharmacol..

[65] Loftsson T. (1995). Effects of cyclodextrins on the chemical stability of drugs in aqueous solutions. Drug Stab..

[66] Grégorio C. (2014). A History of Cyclodextrins. Chem. Rev..

[67] Loftsson T., Hreinsdottir D., Masson M. (2005). Evaluation of cyclodextrin solubilization of drugs. Int. J. Pharm..

[68] Loftsson T., Jarho P., Másson M., Järvinen T. (2005). Cyclodextrins in drug delivery. Expert. Opin. Drug Deliv..

[69] Challa R., Ahuja A., Ali J., Khar R.K. (2005). Cyclodextrins in drug delivery: An updated review. Pharm. Sci. Tech..

[70] Biplab R., Subhadeep S., Koyeli D., Biraj K.B., Swarnab S., Arindam B., Mahendra N.R. (2018). Study to Probe Subsistence of Host-Guest Inclusion Complexes of α and β-Cyclodextrins with Biologically Potent Drugs for Safety Regulatory Dischargement. Sci. Rep..

[71] Loftsson T., Brewster M.E. (1996). Pharmaceutical applications of cyclodextrins. 1. Drug solubilization and stabilization. J. Pharm. Sci..

[72] Loftsson T., Duchene D. (2007). Cyclodextrins and their pharmaceutical applications. Int. J. Pharm..

[73] Ogawa N., Kaga M., Endo T., Nagase H., Furuishi T., Yamamoto H., Kawashima Y., Ueda H. (2012). Quetiapine free base complexed with cyclodextrins to improve solubility for parenteral use. Chem. Pharm. Bull..

[74] Szejtli J. (1998). Introduction and general overview of cyclodextrin chemistry. Chem. Rev..

[75] Marcos L.B. (2015). Strategies to Modify the Drug Release from Pharmaceutical Systems.

[76] Axel F., Stefan B., Sven S., Rolf S. (2004). Degradation of raw or film-incorporated β-cyclodextrin by enzymes and colonic bacteria. Eur. J. Pharm. Biopharm..

[77] Szejtli J. (2004). Past, present and future of cyclodextrin research. Pure Appl. Chem..

[78] Trotta F., Loftsson T., Gaud R.S., Trivedi R., Shende P. (2022). Integration of cyclodextrins and associated toxicities: A roadmap for high quality biomedical applications. Carbohydr. Polym..

[79] Lai W.-F. (2014). Cyclodextrins in non-viral gene delivery. Biomaterials.

[80] Onishi M., Ozasa K., Kobiyama K., Ohata K., Kitano M., Tanigu Chi K., Homma T., Kobayashi M., Sato A., Katakai Y. (2015). Hydroxypropyl-β-cyclodextrin spikes local inflammation that induces Th2 cell and T follicular helper cell responses to the coadministered antigen. J. Immunol..

[81] Tiwari G., Tiwari R., Rai A. (2010). Cyclodextrins in delivery systems: Applications. J. Pharm. Bioallied Sci..

[82] Subrata B., Debi P.N. (2007). Lipid raft disruption by cholesterol depletion enhances influenza A virus budding from MDCK cells. J. Virol..

[83] Annamaria P., Valeriana C. (2015). Role of the lipid rafts in the life cycle of canine coronavirus. J. Gen. Virol..

[84] Yanning L., Ding X.L., James P.T. (2008). Lipid rafts are involved in SARS-CoV entry into Vero E6 cells. Biochem. Biophys. Res. Comm..

[85] Bianka V., Milo M., Tamás S., Szabolcs B. (2022). Molecular interactions in remdesivir-cyclodextrin systems. J. Pharm. Biomed. Anal..

[86] Lajos S., Istvan P., Tamas S. (2021). Sulfobutylether-beta-cyclodextrin-enabled antiviral remdesivir: Characterization of electrospun and lyophilized formulations. Carb. Pol..

[87] Gosselin-Grenet A.S., Mottet-Osman G., Roux L. (2006). From assembly to virus particle budding: Pertinence of the detergent resistant membranes. Virology.

[88] Laliberte J.P., McGinnes L.W., Peeples M.E., Morrison T.G. (2006). Integrity of membrane lipid rafts is necessary for the ordered assembly and release of infectious Newcastle disease virus particles. J. Virol..

[89] Jing L., Fang X., Yujie D., Jiawen Z., Yuan S., Danning L., Natthida S., Jiamiao H. (2022). A Review of Cyclodextrin Encapsulation and Intelligent Response for the Release of Curcumin. Polymers.

[90] Susana S.B., Jéssica S.B., Nádia E.S., Firas E.-S., Filipe A.A. (2021). Cyclodextrins in Antiviral Therapeutics and Vaccines. Pharmaceutics.

[91] Jitendra W., Niranjan G.K., Sonia G., Swetha R., Abhay P., Yury A.R. (2020). Recent Advances in Host–Guest Self-Assembled Cyclodextrin Carriers: Implications for Responsive Drug Delivery and Biomedical Engineering. Adv. Funct. Mater..

[92] European Medicines Agency (2017). Cyclodextrins Used as Excipients (EMA/CHMP/495747/2013).

[93] Cyclodextrin News (2020). Gilead Uses SBECD-Enabled Remdesivir (GS Liqui-Solid 5734) for Treating the First Case of the Novel Coronavirus in the United States.

[94] Goyal G., Vavia P. (2012). Complexation approach for fixed-dose tablet formulation of lopinavir and ritonavir: An anomalous relationship between stability constant, dissolution rate and saturation solubility. J. Incl. Phenom. Macrocycl. Chem..

[95] Sevukarajan M., Bachala T., Nair R. (2010). Novel inclusion complexes of Oseltamivir Phosphate with beta-cyclodextrin: Physicochemical characterization. J. Pharm. Sci. Res..

[96] Rajaram R., Sonaimuthu M., Sekar A., Eun H.C., Fatiha M., Neour L., Yong R.L. (2022). Water-soluble inclusion complexes for a novel anti-viral agent with low toxicity; Oseltamivir with the β-cyclodextrins. J. Mol. Liq..

[97] Rajaram R., Sonaimuthu M., Sekar A., Fatiha M., Neour L., Kuppusamy M., Yong R.L. (2022). A novel and water-soluble material for coronavirus inactivation from oseltamivir in the cavity of methyl and sulfated-β-cyclodextrins through inclusion complexation. J. Pharm. Biomed. Anal..

[98] Arumugam P., Samikannum P., Rajaram R. (2017). Encapsulation of quercetin in β-cyclodextrin and (2-hydroxypropyl)-β-cyclodextrin cavity: In-vitro cytotoxic evaluation. J. Macromol. Sci. A.

[99] Ono A., Freed E.O. (2001). Plasma membrane rafts play a critical role in HIV-1 assembly and release. Proc. Natl. Acad. Sci. USA.

[100] Pickl W.F., Pimentel-Muinos F.X., Seed B. (2001). Lipid rafts and pseudotyping. J. Virol..

[101] Piñeiro A., Pipkin J., Vince Antle V., Garcia-Fandino R. (2022). Remdesivir interactions with sulphobutylether-b- cyclodextrins: A case study using selected substitution patterns. J. Mol. Liq..

[102] Rajaram R., Sundararajulu K., Krishnamoorthy S., Meenakshisundaram M. (2016). Photophysical and photoprototropic characteristics of phenothiazine in aqueous and β-cyclodextrin media. J. Lumin..

[103] Ángel P., James P., Vince A., Rebeca G. (2021). -F.; Aggregation versus inclusion complexes to solubilize drugs with cyclodextrins. A case study using sulphobutylether-β-cyclodextrins and remdesivir. J. Mol. Liq..

[104] Yumeng Z., Zhouming Z., Kai W., Kangjie L., Cai Y., Lin L., Xia S., Tengfei L., Xiaodi G., Haiyan L. (2022). Molecular docking assisted exploration on solubilization of poorly soluble drug remdesivir in sulfobutyl ether-tycyclodextrin. AAPS Open..

[105] Tamas K., Kitti K., Zoltan V., Peter N., Gyorgy P., Florina Z. (2023). Veklury^®^ (remdesivir) for-mulations inhibit initial membrane-coupled events of SARS-CoV-2 infection due to their sulfobutylether-β-cyclodextrin content. Br. J. Pharmacol..

[106] Zhengdong Y., Deqing X., Kah H.J.L., Thomas T., Rita H., Bryan P., Yu-Hui A.F., Eric J., Marsha L.L., Habibi G. (2022). The determination of Sulfobutylether -Cyclodextrin Sodium (SBECD) by LC-MS/MS and its application in remdesivir pharmacokinetics study for pediatric patients. J. Pharm. Biomed. Anal..

[107] Saraswati R.P., Sutriyo Ratika R. (2022). Preparation, Cellular Uptake, and Cytotoxic Evaluation of Remdesivir-Hydroxypropyl-β-Cyclodextrin Inclusion Complex. Biomed. Pharmacol. J..

[108] Robert L.P. (2016). Mouse models of human disease. Evol. Med. Public Health.

[109] Prabu S., Sivakumar K., Kothai Nayaki S., Rajamohan R. (2016). Host-guest interaction of cytidine in β-cyclodextrin microcavity: Characterization and docking study. J. Mol. Liq..

[110] Viswalingam M., Prabu S., Sivakumar K., Rajamohan R. (2016). Spectral characteristics of desipramine in β-cyclodextrin cavity through inclusion complex. J. Macro Sci. Part. A.

[111] Rajamohan R., Kothai Nayaki S., Swaminathan M. (2016). Investigation on association behavior between 1-Aminoisoquinoline and β-Cyclodextrin in solution and solid state. J. Mol. Liq..

[112] Praveena A., Prabu S., Madi F., Rajamohan R. (2023). Theoretical investigation of inclusion complexes of 3-hydroxyflavone andquercetin as guests with native and modified β-cyclodextrins as hosts. Polycycl. Aromat. Compd..

[113] Samuel T., Jones V. (2020). Modified cyclodextrins as broad-spectrum antivirals. Sci. Adv..

[114] Okimoto K., Rajewski R.A., Uekama K., Jona J.A., Stella V.J. (1996). The interaction of charged and uncharged drugs with neutral (HP-beta-CD) and anionically charged (SBE7-beta-CD) beta-cyclodextrins. Pharm. Res..

