# Supplementary material for: Inclusion Complexation of Remdesivir with Cyclodextrins: A Comprehensive Review on Combating Coronavirus Resistance—Current State and Future Perspectives
Source: Molecules. 2024 Oct 9;29(19):4782. doi: 10.3390/molecules29194782 (PMC11477750; doi:10.3390/molecules29194782)
Supplement: Supplementary file 1 [file molecules-29-04782-s001.zip › molecules-3201446-supplementary.pdf]

This review has been compiled based on the results obtained from keyword searches in SciFinder and Google. We selected the results most closely aligned with our search terms and relevant to the focus of the review manuscript, while excluding others.

**S. No**	**Search Keyword**	**Document Type**	**Substance role**	**Duration**	**Database used**	**Exclusion**
**1**	Viral drugs for COVID-19	Journals, and Reviews	Biological applications	2020-2024	SciFinder & Google	The results which are Irrelevant to this review are excluded
**2**	Remdesivir with cyclodextrins	Journals, and Reviews	Biological applications	2019-2024	SciFinder	The results which are Irrelevant to this review are excluded
**3**	About Remdesivir	Journals, and Reviews	Biological study	2000-2024	SciFinder & Google	The results which are Irrelevant to this review are excluded
**4**	About supramolecules	Journals, and Reviews	Biological study	1990-2024	SciFinder & Google	The results which are Irrelevant to this review are excluded
**5**	Other viral drugs with cyclodextrin	Journals, and Reviews	Biological study	2010-2024	SciFinder	The results which are Irrelevant to this review are excluded
